# Analysis of Particle Size and Concentration in Die Sinking Electric Discharge Machining

**DOI:** 10.3390/ma15144932

**Published:** 2022-07-15

**Authors:** Ata ur Rehman, Waseem Arif, Muhammad Imtiaz Hussain, Sajjad Miran, Salman Hussain, Gwi Hyun Lee

**Affiliations:** 1 Department of Industrial Engineering, University of Engineering & Technology Taxila, Taxila 47050, Pakistan; atarehman53@gmail.com (A.u.R.); salman.hussain@uettaxila.edu.pk (S.H.); 2 Department of Mechanical Engineering, University of Gujrat, Gujrat 50700, Pakistan; waseem.arif@uog.edu.pk (W.A.); sajjad.miran@uog.edu.pk (S.M.); 3 Agriculture and Life Sciences Research Institute, Kangwon National University, Chuncheon 24341, Korea; imtiaz@kangwon.ac.kr; 4 Green Energy Technology Research Center, Kongju National University, Cheonan 31080, Korea; 5 Interdisciplinary Program in Smart Agriculture, Kangwon National University, Chuncheon 24341, Korea

**Keywords:** electric discharge machining (EDM), graphite powder, recast layer thickness (RLT), micro hardness, crater size, Box–Behnken design

## Abstract

Electric discharge machining with a powder mix dielectric is a promising technique to harden a work piece’s surface using electricity with a high energy density. The quality of the electrical discharge-machined surface is related to its surface integrity in which the surface’s roughness, residual stresses, micro hardness and surface micro cracks are some of the major factors. In this research, graphite powder was mixed in a dielectric with a particle size of 20 µm, 30 µm, and 40 µm, with the concentration of the graphite powder ranging from 2 g/L to 4 g/L. Moreover, the peak current and pulse time on were also coupled with an additive of graphite powder to investigate the effect on the surface quality, i.e., the recast layer thickness, micro hardness and crater depth as well as the material removal rate (MRR) and tool wear rate (TWR). A Box–Behnken design was employed to design the experiments and the experimental results revealed that the graphite powder size and concentration coupled with the electrical parameters (peak current and pulse time on) significantly influenced the recast layer thickness, micro hardness, crater size, MRR and TWR. The crater depth and micro hardness were maximized at a higher concentration and particle size, while the recast layer thickness was reduced with a higher gain size.

## 1. Introduction

Electric discharge machining (EDM) is one of the fascinating techniques of the non-conventional machining process in which material is removed from a work piece through a series of discharges that take place between the tool and the work part. EDM is generally used for hard materials which are conductive in nature, for very thin materials which cannot be machined using conventional machining and in the die industry for complex geometry creation. EDM is utilized for single tasks or for small batches. This machining mechanism is based on a thermoelectric process in which materials are eroded from a workpiece when controlled sparks are generated from a tool (electrode) [1].

Materials with high hardness values of above 30–35 HRC, e.g., quenched parts, cannot be machined through conventional milling machines; however, materials with high hardness values can be easily machined in EDM processes because the process is independent of the mechanical properties of the material. Recent trends have shown that micro-EDM processes are increasingly needed; for example, the production of delicate products of a small size requires the use of micro-EDM. Micro-EDM can machine micro holes and micro shafts of small sizes of about 5 microns whereas mechanical drilling can drill to only 70 microns while laser machining can reach 40 microns, but micro pins, micro nozzles and complex micro cavities are easily machined using micro-EDM and the water cooling channels in dies and molds also require micro-EDM. Additionally, EDM is used for the machining of WC, SiC, TiC, etc. [2] and recently, research has focused on the following two aspects of EDM machining for ZrB2-based ceramics: the first is ZrB2-constructed ceramics used as electrodes, where the electrode wear is reduced due to a high melting temperature [3]; the second aspect concerns ZrB2-based ceramic workpieces used for extreme temperature applications [4,5]. Yongfeng, Guo et al. [4] utilized ZrB2-SiC ceramics for machining through EDM to identify the material removal rates and optimize the machining parameters for ceramic-based materials. The creation of modern composite materials boosts the applicability of EDM. A study was also conducted by Muller and Monaghan [6] in which a particle-strengthened metal matrix composite was machined via non-conventional machining techniques. The EDM produced a less damaged surface as compared to other processes.

Electric discharge machining with a powder mixed dielectric (PMEDM) is an upgraded technology in the EDM process in which a fine electrical-conductive powder is blended in the dielectric. The working principle of the PMEDM process is described as the proper voltage being applied to an electrode and workpiece that results in an electric field which also charges powder particles added into the dielectric. Due to this, the particles move in a random manner which leads to an improvement in the discharge gap between the electrode and the workpiece. They position themselves to form chains at different spaces during flashing, which act as a bridge in the gap between the electrode and the workpiece. Due to the presence of metal powder particles, the gap voltage and shielding power of the dielectric fluid decrease; thus, short circuiting occurs easily and a series of sparking initiates under the tool [7]. By increasing the frequency of discharging, faster sparking within the discharge occurs and erosion takes place at higher rates on the surface of the workpiece.

The parameters of PMEDM can be categorized on the basis of process parameters and performance parameters. The process parameters are variable and can be controlled to achieve the desired machining performance. For example, an increase in peak current or increase in plasma generation may result in a higher material erosion rate being achieved due to high impulsive forces [8]. A high current value is used for rough machining when a high material removal rate is required while at the same time a high roughness value and tool wear rate is required. Jamadar M. M. and Kavade M.V. [9] used Al powder in a dielectric to assess the effect of the peak current (Ip) and pulse time on (Ton) on the material removal rate (MRR), tool wear rate and surface roughness. It was concluded that a higher material removal rate was obtained at a higher current (14 A) and pulse time on (150 µs), while the concentration of Al in the dielectric was 6 g/L. A low TWR and surface roughness was achieved at a low current (2 A). Dubey et al. [10] used 4 g/L of chromium powder and a particle size of 10–15 microns and claimed that the current and pulse time significantly influenced the MRR. During the pulse off time, the dielectric recovered its strength and molten metal was flushed out from the workpiece. Since in this phase no machining takes place, it must remain shorter to optimize the machining efficiency. A greater duty cycle is described as being when, during the total cycle time, longer sparking takes place which results in a higher MRR; however, increasing the duty cycle places the process in an unstable condition due to poor flushing [11]. In positive polarity, a higher value of energy is produced and a higher MRR is achieved, while a high level of material is removed on the side connected with the positive polarity. The selection of polarity is determined based on the material (workpiece and tool), pulse time on, the current and pulse time off. The polarity of the electrode can be negative or positive. Positive Polarity states that the workpiece is connected to the positive polarity and the tool is connected to negative polarity. Excess material will always be removed from the electrode which is connected to positive polarity. Electrode size and shape are very important factors in PMEDM as the mirror image of the electrode is machined with the workpiece. Therefore, the performance of PMEDM is highly dependent on tool shape configuration. A smaller gap is always set between the electrode and workpiece. This clearance is different for different workpieces, tool material combinations and MRR. Different electrodes used for finish and roughing processes are given in Ref. [12]. The material removal rate (MRR), electrode wear rate (EWR), density of crack and white layer thickness are highly effected by the dielectric type and flushing mechanism [13]. Major flushing techniques which are employed in EDM are given in Ref. [14]. Some of the main purposes of using dielectrics in EDM are to provide shielding when sparking takes place, to provide good flushing and removal of debris from machined surfaces and to provide cooling to the workpiece and tool [15].

The mixing of powders in EDM dielectric enhances the performance of EDM, i.e., increases MRR, decreases TWR and surface roughness (SR) and improves surface characteristics. Different powders have a distinct effect on process performance. Powders added to the dielectric must have electrical conductivity, good suspension capability, thermal conductivity, and should be non-magnetic, non-toxic and neutral [16]. Surface quality with powders blended in an electric discharge machine (PMEDM) is heavily affected by the size of the particle [17]. It has been observed that the gap between the electrode and workpiece increases by increasing the particle size that also increases the roughness value and decreases the material removal rate (MRR) [18]. Tzeng and Lee [19] found that a small particle size (70–80 nm) produced higher MRR and reduced the TWR. M.A. Razak et al. [20] used SiC powder of 10 to 30 microns on Starvax material and found that the machining efficiency increased with optimal particle sizes. MRR increases by increasing the concentration because the discharge increases which ultimately enhances the machining efficiency and reduces surface roughness [21,22]. Kavade et al. [23] used Al powder with concentrations of 1–4 g/L in EDM. It was found that MRR significantly expanded by using Aluminum powder in a dielectric fluid for the roughing stage in EDM. It was also found that MRR is significantly affected by the peak current (Ip) and powder concentration. Saharia N.J et al. [24] used a mixture of both Aluminum and Graphite powder in concentrations of 2, 3 and 4 g/L along with kerosene oil as a dielectric to evaluate the machining attributes of steel EN19 material in electric discharge machining by altering the input parameters of EDM, i.e., Peak current (Ip), Voltage gap (Vg), Aluminum concentration per liter kerosene oil and Graphite concentration per liter kerosene oil. It was found that MRR and TWR decreased. Zain et al. [25] conducted experiments to examine the impact of Tantalum Carbide (TaC) PMEDM on a stainless steel workpiece at several levels of peak current and powder concentrations. The powder concentrations used were 5 g/L, 10 g/L and 15 g/L. The MRR and surface roughness (Ra) were found to improve by increasing the current. However, the performance of the powder concentration was not uniform. The powder density is an important factor as it decides the uniformity of the powder in dielectric. Particles with a lower density are uniformly distributed in machining. Increasing the powder density leads to powder settling at the bottom of the reservoir and as a result the mixing of the powder dielectric becomes redundant [26].

The Material Removal Rate is the amount of material removed during a unit of time. MRR is an important factor used to measure machining efficiency. Every manufacturer considers this as highly important and desires to achieve maximum efficiency. In the EDM process, MRR is very low compared to other conventional machining techniques. A higher MRR and roughness are achieved in the PMEDM of materials with a low melting point [27]. MRR is majorly affected by the following two parameters: peak current (Ip) and pulse time on (Ton). Material erosion during machining relates to tool wear and TWR defines the material eroded in a unit of time. The machining quality is a very important factor to consider. Precision of the machining effect can be achieved by increasing the TWR because the tool is a replica of the desired machining shape. Surface roughness (SR) increases by increasing the energy transfer during sparking. SR is highly dependent on peak current and pulse time on because more heat is transferred to the working part for a longer time which produces a large crater [28]. A good surface finish can be obtained by using a low peak current. The surface integrity is a key feature of surface quality that is produced by the machining process, i.e., value of surface roughness, heat-affected area, recast layer, hardness, cracks and crater size. It consists of mechanical, metallurgical and chemical properties.

Pham Van Dong et al. [29] used the Taguchi–Grey relational analysis to study the optimal combination of process parameters for SKD61 die steel specimens using titanium PMEDM. They discovered that with the selection of optimal machining parameters with PMEDM, superior surface topography can be obtained with increased micro hardness and fewer micro fractures compared to machined specimens. Mohanty S. et al. [30] presented the optimization of MRR with the help of the Taguchi method in the EDM of D2 die Steel. Ishfaq K. et al. [31] studied the potential of nano-graphene powder to upsurge the cutting rate and surface quality in the EDM of Ti alloy. Tran T. H. et al. [32] investigated the main process parameters influencing the surface roughness with SiC powder-mixed EDM of hardened steel. They selected the following PMEDM parameters in their study: pulse time on, pulse time off, powder concentration, pulse current and the server voltage. The effects of mixing electrically conductive carbon nanofibers, semi conductive silicon powder, and insulative alumina powder at different concentrations in a dielectric fluid were studied by observing single discharge craters and the hole machining performance in the EDM of ultrafine particle type tungsten carbide by Gattu S. D. et al. [33].

In die sinker EDM, the quality of the machined surface and performance of the machine mainly depend upon the selected electrical and powder parameters. After conducting a detailed literature review, we focused on four input parameters and five output parameters in this research. Input parameters include conductive powder size, powder concentration, peak current and pulse time on. The band of selected input parameters was derived from the literature review. In friction stir processing, the quality of cast products and mechanical properties depends upon the input parameters selected. After conducting an extensive literature review, we selected four parameters for the current research. Powder size varied from 20 to 40 µm, powder concentration varied from 2 to 4 g/L, pulse time from 15 to 45 µs and peak current changes from 6 to 12 A. All the input parameters were varied in three levels as per the design of the experiment. Saharia, N. J. et al. [24] conducted experiments on steel alloy EN-19 with a peak current of 6–10 A and concentration of powder in the dielectric of 2–4 g/L to check the effect on MRR and TWR. Yoo-Seok Kima, and Chong-Nam Chu [34] studied the tool wear by changing the polarity with graphite powder mixed in dielectric with the concentration ranging from 0.5 to 5 g/L. Hu, F. Q. et al. [35] used a pulse time of up to 12 microsec to check the surface quality, i.e., wear resistance and corrosion resistance. T. Yih-fong and C. Fu-chen [17] investigated the effect of Al, Cr, Cu and SiC powder on recast layer thickness by using powder sizes from 0.7 µ to 100 µ and a pulse time on of 6–75 µs.

In die sinker EDM, the quality of the machined surface and performance of the machine mainly depend upon the selected electrical and powder parameters. Machining efficiency is gauged mainly through MRR, TWR and wear ratio. The quality of the machined surface is gauged through its surface finish and surface integrity, i.e., recast layer thickness, crater morphology, hardness, etc. As EDM is widely used in the mold and die manufacturing industries, mold operational life is heavily dependent on its surface integrity. Thermal fatigue is applied during its operations. A thick recast layer is a potential cause of crack generation. Similarly, hard surfaces produce fine molded parts without defects, i.e., flash, etc. Crater size is very important with respect to surface quality in the mold and die industries. Crater depth defines the flow ability of molten plastic/metal in molds. Deeper craters reduce the flow due to its depth. To overcome this resistance, high injection pressure is applied to inject the plastic in a cavity. This repetitive high pressure reduces the life of a cavity surface due to micro cracks. On the other hand, shallow craters provide the smooth flow of molten plastic/metal inside the mold cavity which leads to a high quality finished product and increases mold life, which ultimately reduces the cost as well.

Most of the available research data describe the effect of powder mixed in dielectric on MRR and TWR. However, the effects of powder size and its concentration coupled with electrical parameters need to be identified on a machined surface with respect to surface integrity, i.e., recast layer thickness, hardness and crater size. These parameters define the surface quality at the microstructure level. These responses ultimately dictate the operational life of mold and die. The Box–Behnken design was employed to design the experiments and the influence of each input parameter on responses was analyzed. This research will assist in the selection of powder and electrical parameters to produce good surfaces without post machining finishing processes to overcome surface defects. The intention of this work was to explore the impact of Graphite powder (different particle size and concentration in die electric) combined with pulse time on and peak current on TWR, MRR, recast layer thickness (RLT), hardness and crater depth in a tool steel workpiece (EN-30B). The main objectives of this work are as follows:

- To quantify the influence of different particle sizes, the concentration of Graphite powder with the variation in pulse time on and pulse current on MRR and TWR;
- To assess the effect of Graphite powder (different particle size with different concentration in dielectric) pulse time on and Peak current on EDMed surface hardness;
- To develop a mathematical model for output responses.

## 2. Experimental Details

### 2.1. Chemical Composition and Material Properties

**Figure 1:**
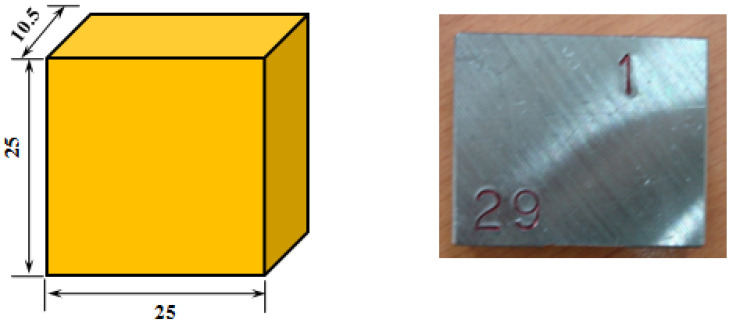
EN-30B alloy steel workpiece sample for experiment 1 and 29 (all dimensions are in mm).

In recent research work, EN-30B alloy steel was used as a workpiece. EN-30B alloy steel is a special steel alloy which can be utilized for high-toughness and high-tensile strength applications. EN-30B alloy steel is also widely used for plastic mold manufacturing. The workpiece was manufactured in a size of 25 × 25 mm^2^ with a thickness value of 10.5 mm as shown in Figure 1.

Furthermore, the chemical composition and mechanical properties of EN-30B alloy steel are outlined in Table 1 and Table 2, respectively.

Graphite powder was used in a mixed form in dielectric to check the desired responses and Copper was selected to manufacture the electrode (tool). A customized tank was built to execute the experimentation with a capacity of 10 L dielectric so that minimum dielectric could be utilized instead of a full tank of the machine. A submersible pump was installed inside the tank close to the workpiece for flushing as well as for stirring purposes. A customized vise was also fabricated to hold the work piece which could be placed in the small tank as well.

### 2.2. Parameter Selection

Graphite powder is used by researchers due to its better efficiency in powder mixed electric machining. Graphite powder was primarily used to optimize the machining efficiency, surface roughness and tool wear rate. Surekha et al. [36] used graphite mixed dielectric and considered the peak current, gap voltage and pulse time on as input process parameters and micro hardness as the response parameter. From the experimental results, a great variation in the surface roughness and micro structure of the machined surface was observed. Mondal et al. [37] used different powders to investigate the effect of process parameters, namely peak current (Ip), gap voltage (Vg) and Pulse time on (Ton) on various responses such as material removal rate (MRR) and surface roughness (SR). A. Kumar et al. (2012) used graphite powder to consider polarity, peak current, pulse time on, gap voltage, powder concentration and duty cycle as input parameters to find the MRR. It was found that MRR increased with the addition of graphite powder. Unses et al. [38] used graphite powder to improve the Electric Discharge Machining (EDM) performance of the Ti-6Al-4V alloy. The EDM performance was quantified by considering MRR, tool electrode wear rate (EWR), relative wear (RW) and surface roughness. With this in mind, we decided to select graphite powder to further investigate the effect of graphite on other parameters of machined surfaces (hardness, recast layer thickness etc.).

The experiment was designed in Design-Expert 7.0 using the Box–Behnken technique. There were four parameters with three levels. The total number of experiments suggested by software was 30 with four parameters. Parameters, along with their levels and the experimental design, are given in Table 3 and Table 4, respectively.

Firstly, 30 samples of EN-30B steel alloy were prepared and markings were engraved on individual samples for identification as shown in Figure 1. The weight of each sample was measured using a digital weighing scale. A customized dielectric container was fabricated with aluminum sheets sized 12″ × 8″ × 10″ (L × W × H) and containers were marked for dielectric level measurements.

A small submersible pump was placed near the sample for flushing as well as for stirring purposes. EDM oil was used as the dielectric and copper electrode was used for the tool. Servo controller was used to maintain the proper discharge gap. Straight polarity was adopted for experimentation. A total of 30 experiments were carried out at different values of particle size, powder concentration, pulse time on and peak current as per DOE. All other electrical and non-electrical parameters were kept constant. Machining time of each experiment was 15 min. After experiments, each sample was carefully packed in a polythene zip bag to keep the machined surface safe.

### 2.3. Response Measurement

After each experiment, the weight of the sample and electrode was measured using a digital weighing scale which can measure up to 0.01 g and MRR and TWR were calculated using Equation (1) and Equation (2), respectively:

(1) $$\mathrm{MRR}=\frac{\mathrm{Wb}-\mathrm{Wa}}{\mathrm{tm}}$$

where Wb is the weight of samples measured before machining, Wa is the weight of samples measured after machining and tm represents machining time.

(2) $$\mathrm{TWR}=\frac{\mathrm{Wbe}-\mathrm{Wae}}{\mathrm{tme}}$$

where Wbe shows the weight of the electrode measured before machining, Wae and tme represent weight of electrode measured after machining and machining time, respectively.

### 2.4. Recast Layer Thickness and Crater Size

Once the pulse ends, dielectric fluid rushes into the gap flushing the molten material from both surfaces (tool and workpiece). However, some material quickly re-solidifies on the surface. This re-solidified layer is called the recast layer. It alters the work piece metallurgical heat affected zone or annealed layer. Surface residual stresses on machined surfaces increase as non-homogeneities within the white layer increase. Such stresses may exceed the fracture strength of the material and result in randomly distributed microcracks on the machined surface. Experiments were conducted at various thicknesses of the workpiece so that recast layer thickness and crater measurements could be measured at the edge. All samples were polished with sand paper of a high number-of-particles. Recast layer thickness and crater size were measured using a scanning electron microscope (SEM). Specifications of the SEM used for measurements are provided in Table 5.

### 2.5. Hardness

Hardness describes a material’s resistance to scratch, indentation and penetration. It is a basic feature of any material. Material hardness is depicted by the hardness number. Material hardness directly correlates with tensile strength. Hardness is a dimensionless number and written as HV. Hardness in this research work was measured using a Vicker’s hardness tester.

## 3. Results and Discussion

This section describes the results derived from the experiments carried out on EDM with four input parameters (graphite powder size, powder concentration, pulse time on, peak current). Five responses (recast layer thickness, micro hardness, crater depth, MRR and TWR) were measured after each experiment. Results of responses were analyzed in Design Expert 7.0 (University of Engineering & Technology, Taxila, Pakistan). An ANOVA of each response was performed and a mathematical model was also derived to determine the response. ANOVA was employed to determine the significance of input parameters on response parameters and is presented in the form of surface plots. ANOVA showed that the input parameters are significant independently as well as in interaction with other input parameters. For example, a Quadratic vs. 2FI statistic model was predicted for microhardness. ANOVA results show that all input parameters were significant along with the interaction of factors (particle concentration and peak current) with a *p*-value of less than 0.0001. The interaction of parameters was also depicted in the response surfaces.

### 3.1. Material Removal Rate (MRR)

The machining process was carried out on the EDM machine at set values of input parameters and 30 samples were machined. The duration of each experiment was 15 min. After every experiment, the MRR of each workpiece was calculated using Equation (1).

The measurements of MRR are provided in Table 6 with the associated input parameters.

The MRR values obtained from the experiments were evaluated using Design-Expert 7.0TM to check the influence of each parameter on MRR and determine the mathematical model for the prediction of response at the input parameters.

**Figure 2:**
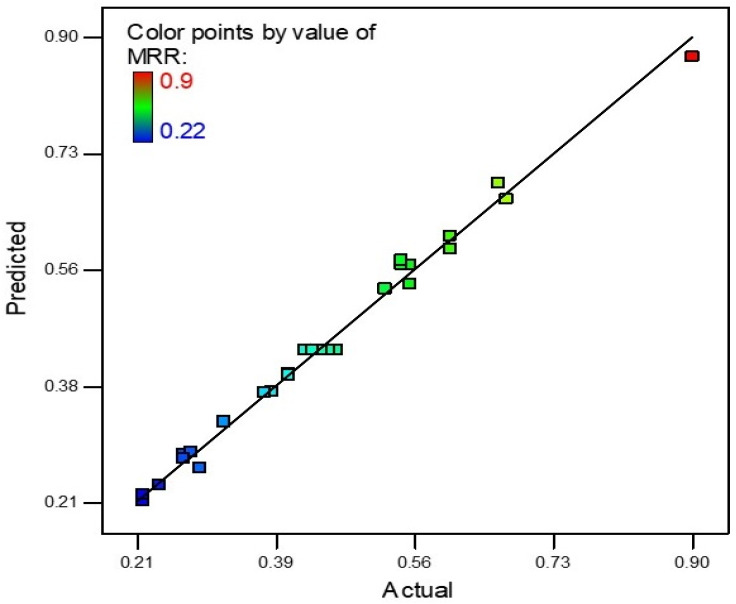
Predicted vs. actual values of MRR.

An MRR mathematical model was firstly designed. An R-square value of near to unity describes the model accuracy. In this scenario, the R-square value was 0.9915 which is very near to unity. The actual and predicted values of MRR are shown in Figure 2. The slope of the line specifies the MRR predicted values and the colored points are actual values that were achieved during experimentation. The model was deemed accurate as most points on the line were near to the predicted values. The theoretical predicted value and experimental results correlate with a maximum 5.88% error for MRR.

**Figure 3:**
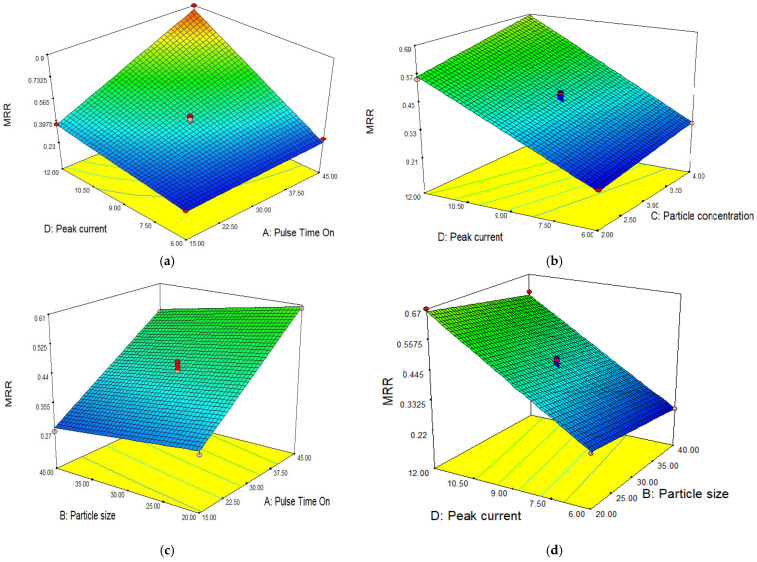

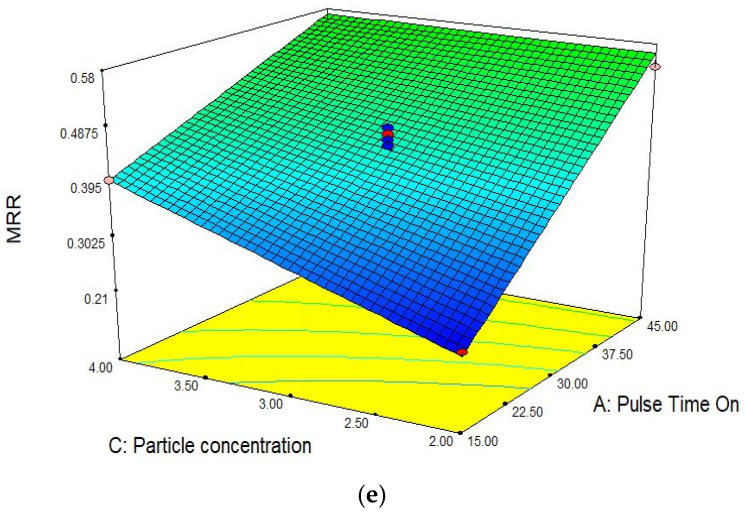
Effect of different parameters on MRR results. (**a**) Peak current vs. pulse time on. (**b**) Peak current vs. particle concentration. (**c**) Particle size vs. pulse time on. (**d**) Peak current vs. particle size. (**e**) Pulse time on vs. particle concentration.

Figure 3 shows the effect of different parameters on MRR. The surface plot shown in Figure 3a illustrates the combined effect of pulse time on and peak current on MRR. It is evident from the plot that by increasing both parameters, an increment in MRR occurred. At a fixed value of peak current, MRR increases by increasing the pulse time on and similarly at a single point of pulse time on, MRR increases with the peak current. However, by increasing both the pulse time on and peak current parameters, MRR significantly increases [10,11,39,40,41]. By increasing the current, more electrons with a higher speed strike which produces high energy and more material melts as a result, which leads to a higher MRR [24]. Furthermore, when pulse time on increase, electrons strike for a longer time to erode the material which ultimately maximizes the MRR. Hence, it is concluded that with the increase in both input parameters at the same time, MRR sharply increases and both parameters are highly influential on MRR. The surface plot in Figure 3b illustrates the combined effect of peak current and particle concentration. It can be noted that the MRR increases with an increase in both the peak current and particle concentration in dielectric [2,11,40,41]. At a fixed value of the peak current, the MRR increased with a higher particle concentration because multiple sparking generation took place at different locations between the tool and workpiece [24]. By increasing the conductive particle concentration, the bridging effect increases and a greater number of sparks are produced which increases the MRR. To obtain a higher MRR, a higher setting of peak current and particle concentration should be established. Furthermore, both parameters are able to increase the MRR. The surface plot in Figure 3c illustrates the combined effect of pulse time on and particle size. It can be observed that MRR increased with an increase in pulse time on and decreased with an increase in particle size. At a fixed value of peak pulse time on, MRR decreased with a large particle size as energy density decreased. Furthermore, at a fixed value of particle size, MRR increased with an increase in pulse time on because as the duration of energy transfer increases with pulse time on, more material melts and higher MRR is achieved. It can be concluded from the surface plot that a higher MRR value is achieved at a higher setting of pulse time on and by lowering the size of particles. The surface plot in Figure 3d explains the combined effect of peak current and particle size. It can be seen that MRR increased with an increase in the peak current and decrease in the particle size of conductive powder. At a fixed value of peak current, MRR decreased with larger particle sizes. MRR is directly influenced by the peak current due to the rate of energy production with a higher setting of peak current. It can be highlighted that particle size is less influential than the peak current on MRR. To obtain optimum MRR, a higher peak current and small particle size are required. In Figure 3e, the surface plot demonstrating the combined effect of pulse time on and particle concentration on MRR is provided. MRR increases with an increase in both parameters. Keeping the same setting of pulse time on, MRR increased with an increase in particle concentration and vice versa. A series of sparking occurs in the presence of a high concentration of powder mixed in dielectric. By simultaneously increasing the powder concentration, a higher value of MRR can be achieved at a higher setting. Both parameters are influential on MRR. Concerning optimal conditions, the maximum MRR was observed at a peak current of 12 A, pulse time on of 45 µs, particle concentration of 4 g/L and particle size of 20 µm.

### 3.2. TWR

Experiments were carried out using an EDM machine at set values of input parameters and 30 samples were machined. The duration of each experiment was 15 min. Copper tool was used as the electrode. After every experiment, TWR was calculated using Equation (2). The measurements of TWR are provided in Table 7 with the associated input parameters.

TWR values obtained from the experiments were evaluated using Design-Expert 7.0.0TM to check the influence of each of the parameters on TWR and determine the mathematical model for the prediction of response with specific input parameters.

**Figure 4:**
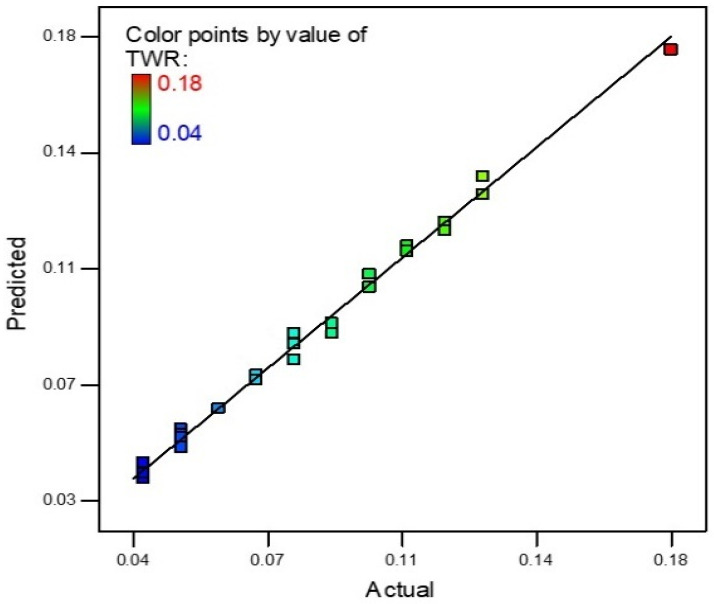
TWR Predicted vs. actual values.

An R-square value near to unity describes model accuracy. In this scenario, the R-square value was 0.9915, which is very near to unity. Actual and predicted values of TWR are shown in Figure 4. The slope of the line specifies the TWR predicted values and the colored points are actual values that were achieved during experimentation. The model was deemed accurate as most points on the line are near to the predicted values. The theoretical predicted value and experimental results correlate with a maximum 7.69% error for TWR.

**Figure 5:**
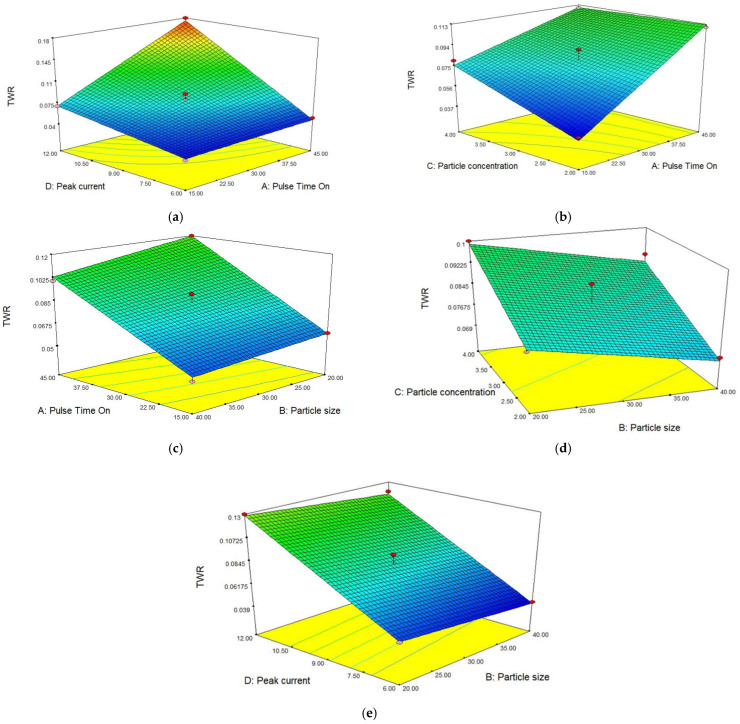
Effect of different parameters on TWR results. (**a**) Peak current vs. pulse time on. (**b**) Pulse time on vs. particle concentration. (**c**) Particle size vs. pulse time on. (**d**) Particle size vs. particle concentration. (**e**) Particle size vs. peak current.

The surface plot shown in Figure 5a illustrates the combined effect of pulse time on and peak current on TWR. It is evident from the plot that by increasing both parameters, an increment in TWR was observed. At a fixed value of peak current, TWR slightly increased by increasing the pulse time on while at a single point of pulse time on, TWR increased with the peak current. However, by increasing both, TWR sharply increased and maximum TWR was observed at a higher setting of peak current and pulse time on [24]. Both factors simultaneously effect the TWR. To reduce the TWR, a low setting of these parameters was required. By increasing the current and pulse time on, more heat was generated, the MRR increased, the tool was also affected by higher temperatures and some material also eroded from the tool as well. Hence, it was concluded that with an increase in both input parameters at the same time, TWR sharply increased and both parameters were found to be highly influential on TWR. In Figure 5b, surface plot demonstrating the combined effect of pulse time on and particle concentration on TWR. TWR increases with an increase in both parameters. Keeping the same setting of pulse time on, TWR increased with an increase in the particle concentration and vice versa [24]. A series of sparks occurs in the presence of a high concentration of powder mixed in dielectric. By simultaneously increasing the powder concentration, a higher value of TWR was achieved at a higher setting similar to MRR. Both parameters were influential on TWR. In Figure 5c, the surface plot shows the combined effect of pulse time on and particle size on TWR. TWR increased with an increase in pulse time on but decreased with increasing particle size. Figure 5d shows the combined effect of particle concentration and particle size. Figure 5e represents the combined effect of peak current and particle size. TWR increases rapidly with increased interactions of the current, pulse duration and graphite particle concentration. TWR has an inverse relationship with particle size.

### 3.3. Micro Hardness

Micro hardness was measured for each sample with a Vickers hardness tester. The measurements of micro hardness are provided in Table 8 with the associated input parameters.

Micro hardness values obtained from the experiments were evaluated using Design-Expert 7.0.0TM to check the influence of each parameter on hardness and determine the mathematic model for prediction of response at the input parameters.

**Figure 6:**
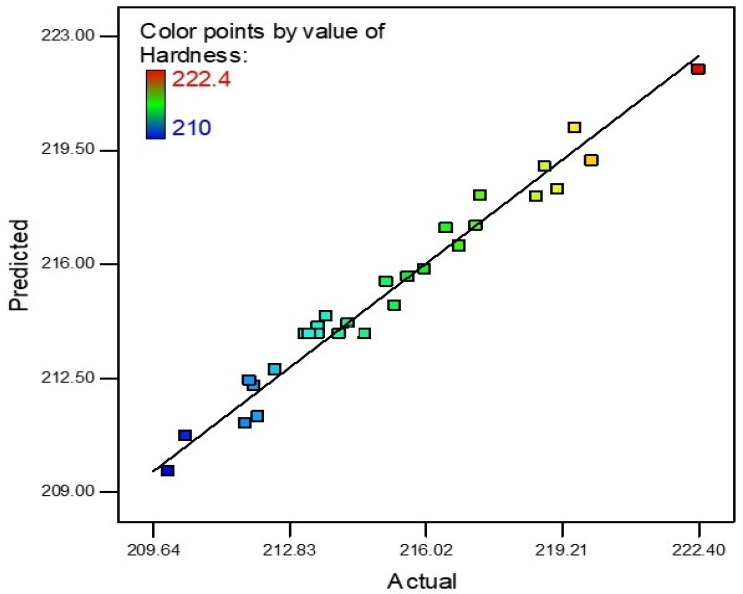
Micro hardness predicted vs. actual.

An R-square value of near to unity describes model accuracy. In this scenario, the R-square value was 0.9688. Actual and predicted values of micro hardness are shown in Figure 6. The slope of the line specifies the hardness predicted values and the colored points are actual values that are achieved after experimentation. The model is deemed accurate as most points on the line are near to the predicted values. The theoretical predicted value and experimental results correlate with a maximum 5.29% error for micro hardness.

**Figure 7:**
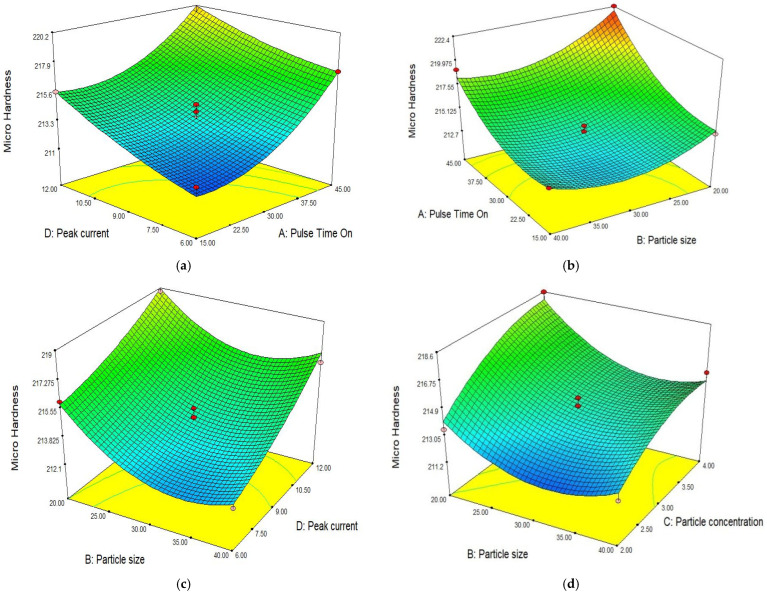

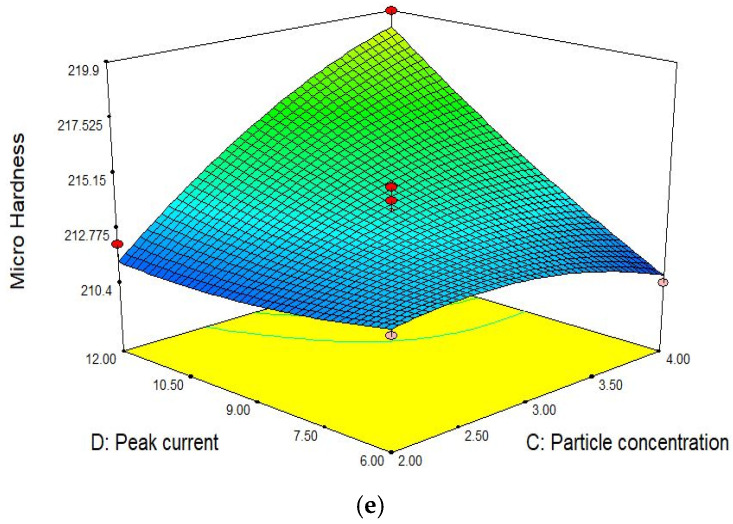
Effect of different parameters on microhardness. (**a**) Peak current vs. pulse time on. (**b**) Particle size vs. pulse time on. (**c**) Particle size vs. peak current. (**d**) Particle size vs. particle concentration. (**e**) Particle concentration vs. peak current.

In Figure 7a, the surface plot shows the relation between peak current and pulse time on. It is clear from the surface plot that micro hardness increased with an increase in the input parameters [10]. The micro hardness exponential increased with the peak current and pulse time on [36]. At a constant point of the peak current, the microhardness increased with pulse time on. At a higher pulse time on, more heat transfer occurred to the workpiece for a longer time. Similarly, by increasing the peak current, electron strikes produced the maximum temperature. When the peak current and pulse time on increased simultaneously, the heating effect coupled with heat transfer to the workpiece and material quench in oil occurred quickly, which led to an increase in the micro hardness. In Figure 7b, the surface plot illustrates the effect of pulse time on and particle size on micro hardness. Both factors significantly influenced the response. At a lower setting of pulse time on, the microhardness was low and when the pulse time increased at a constant particle size, micro hardness increased sharply. On the other hand, it can be noted that microhardness experienced a reverse effect by particle size. At a higher particle size, microhardness was low due to lower MRR and the transfer of energy. By increasing the pulse time on and reducing the particle size, microhardness exponentially increased and maximum hardness was achieved at a higher setting of pulse time on and with small particle sizes. In Figure 7c, the surface plot illustrates the effect of peak current and particle size on micro hardness. Both factors significantly influenced the response. Particle size is more influential than peak current. At a lower setting of the peak current, microhardness is low and when increasing the pulse time at a constant particle size, micro hardness increases gradually. On the other hand, it can be noted that microhardness experienced a reverse effect by particle size. At a higher particle size, microhardness was low due to the lower MRR and transfer of energy. At a small particle size, the number of sparks generated increases, which ultimately leads to a greater temperature rise. By increasing the current and reducing the particle size, microhardness exponentially increases and the maximum hardness is achieved at a higher setting of pulse time on and small particle size. The surface plot in Figure 7d describes the significance of particle size and powder concentration in dielectric on microhardness. Microhardness sharply increases with particle concentration [3], while hardness initially reduces when reducing the particle size then suddenly increases exponentially with a reduction in the size of particles. Higher hardness was achieved at large powder concentrations and small particle sizes. The surface plot in Figure 7e illustrates that micro hardness sharply increased with increases in the particle concentration and peak current. By increasing this individual parameter, the increase in microhardness was small but the due to the interaction of both effects, a maximum increment was observed. High hardness is recommended for toughness and it is a function of heat transfer to the workpiece during machining and subsequently it quenches in the dielectric medium. High hardness (222.8 HV) is observed at 45 µs pulse time on, particle size 20 µm. A similar peak current 12 A is recommended for optimal hardness, while a particle concentration of 3 g/L is recommended.

### 3.4. Recast Layer Thickness (RLT)

**Figure 8:**
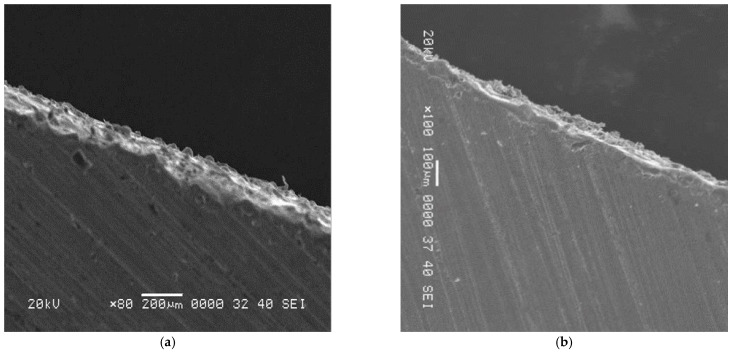
SEM graphs of RLT. (**a**) Highest RLT 68.5 µm observed at particle size of 20 µm and peak current 12 A. (**b**) Minimum RLT 38.7 µm observed at particle size of 20 µm and peak current 9 A.

Recast layer thickness was measured on each sample using a scanning electron microscope (SEM) as shown in Figure 8a,b. The measurements of the micro recast layer are provided in Table 9 with the associated input parameters.

The recast layer thickness values obtained from the experiments were evaluated using Design-Expert 7.0.0TM to check the influence of each parameter on recast layer thickness and to determine the mathematical model for prediction of the response at input parameters.

**Figure 9:**
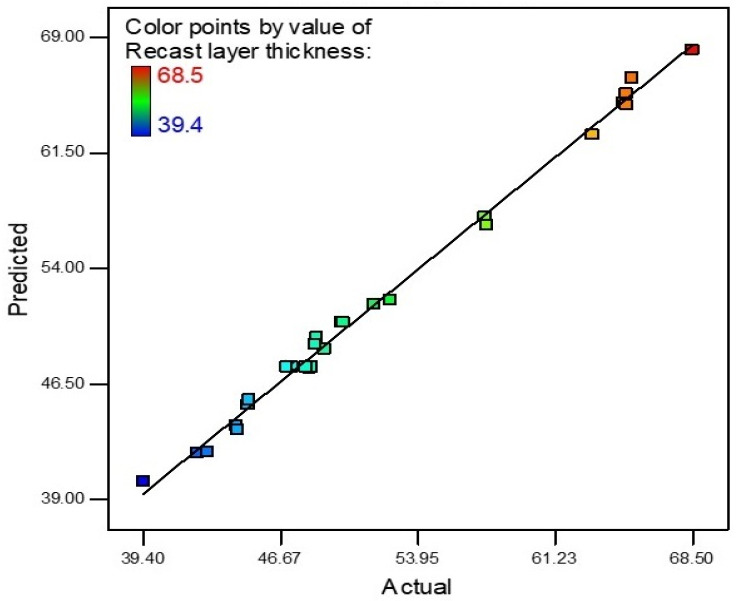
RLT predicted vs. actual values.

An R-square value near to unity describes the model accuracy. In this scenario, the R-square value was 0.9950. The actual and predicted values of RLT are shown in Figure 9. The slope of the line specifies the RLT predicted values and the colored points are actual values that were achieved after experimentation. The model is deemed accurate as most points on the line are near to the predicted values. The theoretical predicted value and experimental results correlate with a maximum 5.91% error for RLT.

**Figure 10:**
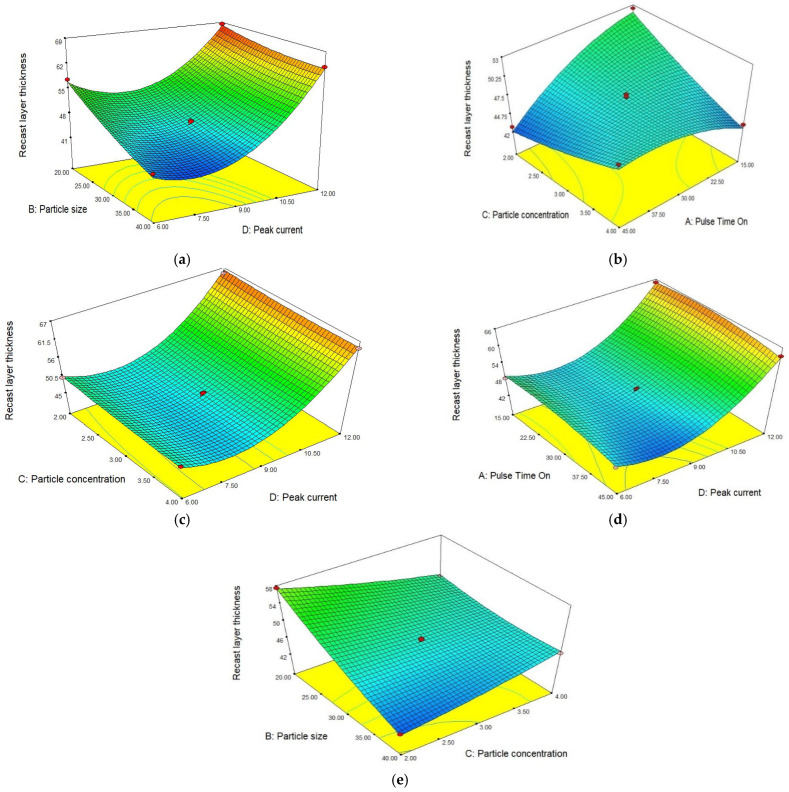
Effect of different parameters on RLT. (**a**) Peak current vs. particle size. (**b**) Pulse time on vs. particle concentration. (**c**) Particle concentration vs. peak current. (**d**) Peak current vs. pulse time on. (**e**) Particle size vs. particle concentration.

In Figure 10a, the surface plot demonstrates the effect of particle size and peak current on recast layer thickness. The recast layer thickness significantly altered for the input parameters. At a constant peak current, particle size significantly modifies the recast layer thickness. The recast layer thickness increases with decreased particle sizes [19]. The maximum RLT was achieved at a particle size of 20 µm. On the other hand, RLT increased with an increased peak current [9,35]. Initially, RLT decreased at low values then exponentially increased with a growth in the current. After increasing the current value and decreasing the particle size, a large increment was observed in RLT. The surface plot in Figure 10b shows that pulse time on and particle concentration have a significant effect on the RLT. It can be noted from the surface plot that individual parameters influence the RLT to a lesser degree. However, with the interaction of both parameters, a significant increase in RLT was observed. By increasing the concentration, more sparks were generated; similarly, with a longer pulse duration more energy density was found for the workpiece. Subsequently, more plasma was generated and thick RLT was produced [3,35]. Figure 10c shows the combined effect of particle concentration and peak current on RLT. The surface plot in Figure 10d represents the pulse time on and peak current. Similarly, Figure 10e demonstrates the effect of particle size and particle concentration on RLT. Two parameters, namely peak current and particle size, are more influential on RLT as compared to other parameters. RLT is recommended to be as small as possible. A small RLT was observed at a particle size of 40 µm, particle concentration of 2 g/L, peak current of 8 A, and pulse time on has a relationship with particle size. A 39.76 µm RLT was observed at a particle size of 40 µm and pulse time on 45 µs.

### 3.5. Crater Depth

The crater depth was measured for each sample using a Scanning Electron Microscope (SEM). The measurements of craters are provided in Table 10 with the associated input parameters.

Crater Depth values obtained from SEM measurements were evaluated using Design-Expert 7.0.0TM to check the influence of each parameter on hardness and determine the mathematic model for the prediction of the response at the input parameters.

**Figure 11:**
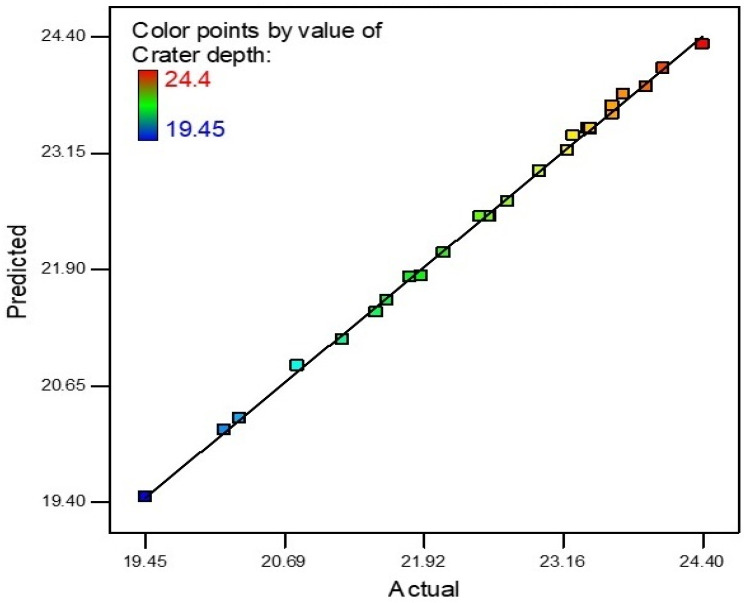
Crater depth predicted vs. actual values.

An R-square value near to unity describes model accuracy. In this scenario, the R-square value was 0.9987. The actual and predicted values of the crater depth are shown in Figure 11. The slope of the line specifies the crater depth predicted values and the colored points are actual values that were achieved after experimentation. The model was deemed accurate as most points on the line are near to the predicted values. The theoretical predicted value and experimental results correlate with the maximum 5.31% error for crater depth.

**Figure 12:**
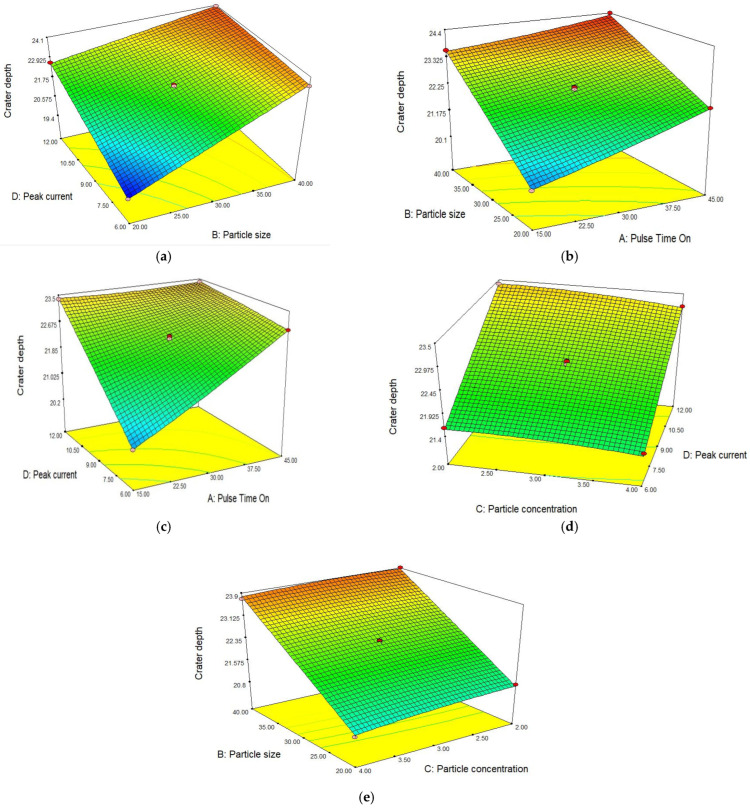
Effect of different parameters on craters depth, (**a**) Peak current vs. particle size, (**b**) Pulse time on vs. particle size, (**c**) Pulse time on vs. peak current, (**d**) Peak current vs. particle concentration, (**e**) Particle size vs. particle concentration.

In Figure 12a, the surface plot demonstrates the effect of particle size and peak current on craters produced as the result of each spark. At a low current, spark strength was low and less material was removed. Due to this, small sized craters were produced. Similarly, small sized particles produced multiple sparks on the workpiece surface as the material was removed equally and craters were therefore smaller in size. From the surface plot, it can be noted that a large crater size was produced at a higher value of peak current [10] and with a higher particle size. To reduce the crater depth, low current and small sized particles are required to be selected. The surface plot in Figure 12b shows that crater depth is highly sensitive to pulse time on and particle size. Both significantly affect the response. By increasing the pulse time on, the time of pulse extended due to which more energy transferred to the workpiece and materials melted more at the pulse strike area. As a result of this, a deep crater was produced. Similarly, with small sized particles, sparks were equally generated all over the surface and almost the complete surface are experienced erosion. Due to the equal distribution of sparks, a more finished surface was produced. By increasing the particle size, spark generation reduced and more craters were produced. The surface plot demonstrates that more craters were produced after increasing the pulse time on and particle size. At higher values of the said parameters, a deeper crater was produced and the roughness increased accordingly. In Figure 12c, the surface plot depicts that both peak current and pulse time on directly affected the response. Crater size increased with an increase in both the input parameters. By increasing the values simultaneously, the crater size sharply increased accordingly. To obtain a smooth surface, a lower setting of peak current and pulse time on is recommended. The surface plot in Figure 12d shows that crater size mainly depends on peak current. The significance of particle concentration in dielectric is far less than the significance of peak current. Crater size slightly reduces with an increase in the concentration. High crater depression is found at a higher current and low particle concentration. Figure 12e represents the particle size and particle concentration effect on crater depth. Crater depth increased with increased values of three parameters, namely particle size, pulse time on and peak current. A shallow crater is recommended for the production of a good surface. Shallow craters are produced at a 15 µs pulse time on, particle size of 20 µm and peak current of 6 A. However, particle concentration is much less influential on crater size.

## 4. Conclusions

The effects of graphite powder, particle size and concentration in dielectric with a combination of pulse time on and peak current on MRR, TWR, RLT, hardness and crater depth of the steel tool workpiece (EN-30B) were investigated in the current study. ANOVA was employed to determine the effect of the input parameters on response parameters. ANOVA showed that the parameters are significant independently as well as in interaction with other input parameters. It was observed from the current study that MRR increases with increased current settings, pulse duration, graphite particle concentration and with a small particle size. The maximum MRR was observed at a peak current of 12 A, pulse time of 45 µs, particle concentration of 4 g/L and particle size of 20 µm. TWR sharply increased with an increase in the interaction of the current setting, pulse duration and graphite particle concentration. TWR had an inverse relationship with the particle size. Similarly, microhardness increased with higher values of pulse time on, peak current and powder particle concentration. High hardness (222.8 HV) was observed at 45 µs pulse time on and at a particle size of 20 µm. A peak current of 12 A is recommended for optimal hardness. Two parameters, namely peak current and particle size, are more influential on RLT as compared to other parameters. The small RLT was observed at a particle size of 40 µm, particle concentration of 2 g/L and peak current of 8 A. RLT of 39.76 µm was observed at a particle size of 40 µm and pulse time on of 45 µs. The crater depth is a subset of surface roughness. A similar effect was found for crater depth as that of surface roughness, which was observed by a variation in the parameters. Crater depth increased with greater particle size, pulse time on and peak current. Shallow craters were produced at 15 µs pulse time on, a particle size of 20 µm and peak current of 6 A. However, particle concentration is much less influential on crater size. The theoretically predicted values and experimental results of all the response parameters correlated very well and the error percentage was less than 8%.

## Figures and Tables

**Table 1:** Chemical Composition of EN-30B alloy steel (Weight %).

Chemical Name	C	Si	Mn	Ni	Cr	Mo	P	S
**Min. (%)**	0.26	0.10	0.45	3.90	1.10	0.20	-	-
**Max. (%)**	0.34	0.35	0.70	4.30	1.40	0.35	0.025	0.025

**Table 2:** Mechanical properties of EN-30B alloy steel (Weight %).

Property	Yield Strength	Tensile Strength	Elongation	Hardness
**Value**	154 MPa	234 MPa	56%	85 HRB

**Table 3:** Parameters and Levels used in the current study.

Parameters	Unit	Levels		
		Low	Medium	High
**Graphite Powder Size**	µm	20	30	40
**Powder Concentration**	g/L	2	3	4
**Peak Current**	A	6	9	12
**Pulse Time On**	µs	15	30	45

**Table 4:** Design of Experiments performed in the current study.

Run	Pulse Time On (µs)	Particle Size (µm)	Concentration (g/L)	Peak Current (A)
1	30	30	2	6
2	30	30	3	9
3	30	30	4	6
4	45	30	4	9
5	30	40	3	12
6	30	40	4	9
7	30	20	3	12
8	15	20	3	9
9	45	40	3	9
10	30	30	3	9
11	30	40	2	9
12	45	20	3	9
13	30	30	3	9
14	30	30	2	12
15	45	30	3	6
16	30	30	3	9
17	30	30	3	9
18	30	20	2	9
19	30	30	4	12
20	15	30	2	9
21	15	30	3	12
22	30	20	3	6
23	30	40	3	6
24	30	30	3	9
25	15	30	3	6
26	45	30	3	12
27	30	20	4	9
28	15	40	3	9
29	45	30	2	9
30	15	30	4	9

**Table 5:** Specification of scanning electron microscope used in the recent research work.

Magnification	8× to 300,000×
Resolution	3.0 nm
Detectors	SEI and BEI
Probe Current	1 pA to Maximum 1 µA
Filament	Pre-centered Tungsten Hairpin
Display system	Display tube 17″ monitor
Accelerating Voltage	0.3 to 30 kV (55 steps)
Vacuum System Control	Fully automatic
Vacuum Ultimate Pressure	0.1 mPa order

**Table 6:** MRR results with different parameters.

Run	Pulse Time On (µs)	Particle Size (µm)	Concentration (g/L)	Peak Current (A)	MRR (g/min)
1	30	30	2	6	0.22
2	30	30	3	9	0.42
3	30	30	4	6	0.28
4	45	30	4	9	0.54
5	30	40	3	12	0.6
6	30	40	4	9	0.45
7	30	20	3	12	0.67
8	15	20	3	9	0.32
9	45	40	3	9	0.52
10	30	30	3	9	0.45
11	30	40	2	9	0.37
12	45	20	3	9	0.6
13	30	30	3	9	0.44
14	30	30	2	12	0.55
15	45	30	3	6	0.29
16	30	30	3	9	0.43
17	30	30	3	9	0.44
18	30	20	2	9	0.40
19	30	30	4	12	0.66
20	15	30	2	9	0.22
21	15	30	3	12	0.38
22	30	20	3	6	0.27
23	30	40	3	6	0.22
24	30	30	3	9	0.46
25	15	30	3	6	0.24
26	45	30	3	12	0.90
27	30	20	4	9	0.55
28	15	40	3	9	0.27
29	45	30	2	9	0.54
30	15	30	4	9	0.40

**Table 7:** TWR results by using different parameters.

Run	Pulse Time On (µs)	Particle Size (µm)	Concentration (g/L)	Peak Current (A)	TWR (g/min)
1	30	30	2	6	0.04
2	30	30	3	9	0.08
3	30	30	4	6	0.05
4	45	30	4	9	0.11
5	30	40	3	12	0.12
6	30	40	4	9	0.09
7	30	20	3	12	0.13
8	15	20	3	9	0.06
9	45	40	3	9	0.1
10	30	30	3	9	0.09
11	30	40	2	9	0.07
12	45	20	3	9	0.12
13	30	30	3	9	0.08
14	30	30	2	12	0.11
15	45	30	3	6	0.05
16	30	30	3	9	0.08
17	30	30	3	9	0.08
18	30	20	2	9	0.08
19	30	30	4	12	0.13
20	15	30	2	9	0.04
21	15	30	3	12	0.07
22	30	20	3	6	0.05
23	30	40	3	6	0.04
24	30	30	3	9	0.09
25	15	30	3	6	0.04
26	45	30	3	12	0.18
27	30	20	4	9	0.1
28	15	40	3	9	0.05
29	45	30	2	9	0.11
30	15	30	4	9	0.08

**Table 8:** Vickers micro hardness results.

Run	Pulse Time On (µs)	Particle Size (µm)	Concentration (g/L)	Peak Current (A)	Micro Hardness (HV)
1	30	30	2	6	212
2	30	30	3	9	213.3
3	30	30	4	6	210.4
4	45	30	4	9	217.3
5	30	40	3	12	216.5
6	30	40	4	9	215.3
7	30	20	3	12	218.8
8	15	20	3	9	215.1
9	45	40	3	9	219.1
10	30	30	3	9	213.2
11	30	40	2	9	211.9
12	45	20	3	9	222.4
13	30	30	3	9	213.5
14	30	30	2	12	212.1
15	45	30	3	6	217.2
16	30	30	3	9	214
17	30	30	3	9	214.6
18	30	20	2	9	213.5
19	30	30	4	12	219.9
20	15	30	2	9	210
21	15	30	3	12	215.6
22	30	20	3	6	216
23	30	40	3	6	212.5
24	30	30	3	9	214.6
25	15	30	3	6	211.8
26	45	30	3	12	219.5
27	30	20	4	9	218.6
28	15	40	3	9	214.2
29	45	30	2	9	216.8
30	15	30	4	9	213.7

**Table 9:** Recast layer thickness results.

Run	Pulse Time On (µs)	Particle Size (µm)	Concentration (g/L)	Peak Current (A)	Recast Layer Thickness (µm)
1	30	30	2	6	50
2	30	30	3	9	48
3	30	30	4	6	49
4	45	30	4	9	48.2
5	30	40	3	12	65
6	30	40	4	9	47.05
7	30	20	3	12	68.5
8	15	20	3	9	51.62
9	45	40	3	9	39.4
10	30	30	3	9	47.1
11	30	40	2	9	42.25
12	45	20	3	9	49.9
13	30	30	3	9	48.3
14	30	30	2	12	65.3
15	45	30	3	6	45
16	30	30	3	9	47
17	30	30	3	9	47.27
18	30	20	2	9	57.5
19	30	30	4	12	65
20	15	30	2	9	52.5
21	15	30	3	12	64.83
22	30	20	3	6	57.6
23	30	40	3	6	44.4
24	30	30	3	9	48
25	15	30	3	6	48.6
26	45	30	3	12	63.2
27	30	20	4	9	48.5
28	15	40	3	9	44.95
29	45	30	2	9	42.8
30	15	30	4	9	44.33

**Table 10:** Measurement of crater depth.

Run	Pulse Time On (µs)	Particle Size (µm)	Concentration (g/L)	Peak Current (A)	Crater Depth (µm)
1	30	30	2	6	21.6
2	30	30	3	9	22.5
3	30	30	4	6	21.5
4	45	30	4	9	22.95
5	30	40	3	12	24.05
6	30	40	4	9	23.7
7	30	20	3	12	22.67
8	15	20	3	9	20.15
9	45	40	3	9	24.4
10	30	30	3	9	22.51
11	30	40	2	9	23.9
12	45	20	3	9	22.1
13	30	30	3	9	22.5
14	30	30	2	12	23.4
15	45	30	3	6	22.95
16	30	30	3	9	22.42
17	30	30	3	9	22.46
18	30	20	2	9	21.2
19	30	30	4	12	23.2
20	15	30	2	9	21.8
21	15	30	3	12	23.38
22	30	20	3	6	19.45
23	30	40	3	6	23.6
24	30	30	3	9	22.45
25	15	30	3	6	20.29
26	45	30	3	12	23.4
27	30	20	4	9	20.8
28	15	40	3	9	23.6
29	45	30	2	9	23.25
30	15	30	4	9	21.9

## Data Availability

Not applicable.
